# Approximate Arithmetic Training Improves Informal Math Performance in Low Achieving Preschoolers

**DOI:** 10.3389/fpsyg.2018.00606

**Published:** 2018-05-15

**Authors:** Emily Szkudlarek, Elizabeth M. Brannon

**Affiliations:** Department of Psychology, University of Pennsylvania, Philadelphia, PA, United States

**Keywords:** preschool math, approximate number system, cognitive training, approximate arithmetic, numerical cognition, tablet application

## Abstract

Recent studies suggest that practice with approximate and non-symbolic arithmetic problems improves the math performance of adults, school aged children, and preschoolers. However, the relative effectiveness of approximate arithmetic training compared to available educational games, and the type of math skills that approximate arithmetic targets are unknown. The present study was designed to (1) compare the effectiveness of approximate arithmetic training to two commercially available numeral and letter identification tablet applications and (2) to examine the specific type of math skills that benefit from approximate arithmetic training. Preschool children (*n* = 158) were pseudo-randomly assigned to one of three conditions: approximate arithmetic, letter identification, or numeral identification. All children were trained for 10 short sessions and given pre and post tests of informal and formal math, executive function, short term memory, vocabulary, alphabet knowledge, and number word knowledge. We found a significant interaction between initial math performance and training condition, such that children with low pretest math performance benefited from approximate arithmetic training, and children with high pretest math performance benefited from symbol identification training. This effect was restricted to informal, and not formal, math problems. There were also effects of gender, socio-economic status, and age on post-test informal math score after intervention. A median split on pretest math ability indicated that children in the low half of math scores in the approximate arithmetic training condition performed significantly better than children in the letter identification training condition on post-test informal math problems when controlling for pretest, age, gender, and socio-economic status. Our results support the conclusion that approximate arithmetic training may be especially effective for children with low math skills, and that approximate arithmetic training improves early informal, but not formal, math skills.

## Introduction

Early math competency is an important predictor of later academic achievement and a variety of measures of adult health and economic well-being (Duncan et al., 2007; Jordan et al., 2009, 2010; Reyna et al., 2009; Geary et al., 2013; Gerardi et al., 2013). It is critical that children enter kindergarten and first grade prepared to embark on formal math learning, however, there is wide variation in the level of math skill children acquire during the preschool years (Jordan et al., 2006). Conceptual knowledge of addition and subtraction is an especially important skill for children at the beginning of formal math education (Nunes et al., 2007; Ching and Nunes, 2017). Therefore, improving early conceptual knowledge of arithmetic is an important way to enhance math readiness in preschool children.

The Approximate Number System (ANS) supports an intuitive sense of number that allows adults, human infants, and many non-human animals to compare, estimate, and manipulate non-symbolic and approximate numerical quantities (Feigenson et al., 2004). For example, the ANS allows children to distinguish which of two sets of objects is greater in number. There is a modest but significant relation between ANS acuity and symbolic math skills (see Chen and Li, 2014; Fazio et al., 2014; Schneider et al., 2016 for meta-analyses). Specifically, children and adults with greater ANS acuity score better on math achievement measures such as the TEMA, the calculation portion of the Woodcock Johnson, or even self-reported SAT exams (Halberda et al., 2008, 2012). This relation suggests that the ANS may be a building block upon which children anchor their concept of symbolic number. Previous research has demonstrated that children can solve math problems non-symbolically and approximately before they comprehend the same operations symbolically (Barth et al., 2005). With the ANS, young children can compare, add, subtract, multiply, and divide, and solve simple linear equations using sets of objects with ratio-dependent precision (Barth et al., 2006; McCrink and Spelke, 2010, 2016; Kibbe and Feigenson, 2015). In contrast to these prodigious non-symbolic and approximate mathematical abilities, children must be explicitly taught how to solve the same symbolic mathematical problems effectively over years of formal schooling.

To further test the hypothesis that ANS representations serve as a building block for symbolic mathematics, recent work has tested the possible causal relation between ANS based tasks and symbolic math skills. In the first of these studies, Park and Brannon (2013, 2014) trained adults on an approximate arithmetic task and tested their symbolic arithmetic fluency before and after training. During approximate arithmetic training, subjects watched addition and subtraction events depicted with animated arrays of dots. For example, during an addition trial, an array of dots appeared and then moved behind an opaque box. A second array of dots then appeared and also moved behind the box. After watching this animation, the subject imagines the sum behind the box and compares this imagined quantity to a second visible quantity. Adults trained on this approximate arithmetic task showed greater improvement on a symbolic arithmetic assessment compared to a no contact control group, a group trained on general knowledge facts, a group trained to rapidly order numerals, a group trained on a visuo-spatial short term memory task, and a group trained on approximate numerosity comparisons. Thus, for adults, practice mentally manipulating approximate quantities in arithmetic operations yielded a benefit for symbolic arithmetic performance that was not afforded by any of the control training tasks. This finding raised the important question as to whether non-symbolic and approximate arithmetic training could also be effective for children. If shown to be effective for preschoolers, approximate arithmetic training could be a useful tool for introducing arithmetic concepts to children before they are ready to master symbolic arithmetic in the classroom.

A handful of experiments have explored this possibility by training children on approximate arithmetic tasks and testing their symbolic math abilities after training (Hyde et al., 2014; Khanum et al., 2016; Park et al., 2016; Dillon et al., 2017). Hyde et al. (2014) found that first grade children who completed a session of approximate arithmetic or dot comparison training were faster at completing a symbolic arithmetic test than children who had completed a training session of line length addition or brightness comparison. This finding was replicated in an independent sample of children, suggesting that approximate arithmetic training improves arithmetic fluency (Khanum et al., 2016). In a large scale study conducted in India, approximate arithmetic combined with geometry training improved non-symbolic but not symbolic math performance in preschool and elementary school children (Dillon et al., 2017). Children who participated in the non-symbolic math training condition maintained higher non-symbolic math skills 1 year after training compared to the children in the control group. Park et al. (2016) tested the effectiveness of approximate arithmetic training with preschool children using a pre/post test training paradigm. An approximate arithmetic tablet application called Max's Math Game was created to mirror the adult approximate arithmetic training studies of Park and Brannon (2013, 2014). Over 10 training sessions preschool aged children played Max's Math Game or a non-math picture-memory game. Children were tested with The Third Edition of the Test of Early Mathematics Achievement (TEMA-3; Ginsburg and Baroody, 2003), and with measures of vocabulary, short term memory, and executive function before and after training. Preschoolers who trained on the approximate arithmetic task selectively improved on the TEMA-3 significantly more than children who trained on the picture-memory game. Taken together, the research on non-symbolic math training suggests that practice with approximate and non-symbolic arithmetic may be an effective way to improve the math skills of young children (but see Szucs and Myers, 2017).

The current study aims to advance approximate arithmetic training research in two ways. First, the current study was designed to provide insight into the nature of the symbolic math skills that approximate arithmetic training benefits. Prior research has found that ANS acuity correlates with TEMA-3 questions that assess informal, but not formal, math abilities (Libertus et al., 2013). Thus, it is possible that approximate arithmetic training selectively improves informal, but not formal, symbolic math abilities. Informal math abilities include counting, assessments of numerical magnitude, and knowledge of the ordinal relationship between numbers in the counting sequence, while formal math abilities include fact retrieval and numeral identification (Ginsburg and Baroody, 2003; Jordan et al., 2009). Informal symbolic math skills require children to use number words and symbols in mathematical operations. For example, the informal math question “You have 4 pennies. I give you 2 more pennies. How many pennies do you have altogether?” is a conceptual test of addition. In contrast, formal math skills involve the memorization of math facts. For example, when a child is shown the numeral “4” and asked “What number is this?” the child must recall that the symbol “4” corresponds to the word “four.” During approximate arithmetic training children do not gain experience with the formal math skill of identifying that the symbol “4” corresponds to the word “four,” however, the process of addition is modeled repeatedly. Thus, approximate and non-symbolic practice with addition and subtraction may induce improved performance selectively on informal math problems that test knowledge of arithmetic concepts. To test this hypothesis in the current study, we created a measure of early math skills inspired by the Number Sense Screener (NSS; Research Edition: Glutting and Jordan, 2012). Many standardized tests of math for young children, like the TEMA-3, are good measures of general early math performance, but due to age standardization and titration procedures it is difficult to break down the specific math skills improved by training. Our measure is split into sections, with each section defined by a specific math skill. This design allowed us to separately evaluate improvements in informal and formal math skills as a result of approximate arithmetic training.

The second aim of the current study was to compare the effectiveness of approximate arithmetic training to existing math educational practices. Specifically, we compared approximate arithmetic training to two commercially available applications designed to improve symbol knowledge, the 123 Ninja and ABC Ninja games (alligatorapps.com). Previous studies have compared the effectiveness of approximate arithmetic training to control groups trained with non-numerical tasks, and not to educationally relevant math games. For approximate arithmetic to be useful in a classroom, it should be at least as effective as other age appropriate math games. In the control training games used in the current study, children see multiple numerals (123 Ninja) or letters (ABC Ninja) floating across the screen. The child then hears one letter or one number word and is tasked with selecting the appropriate symbol. Educational tablet applications have gained popularity in recent years, but they have been largely untested for their actual educational outcomes (Hirsh-Pasek et al., 2015). We included the 123 Ninja game to assess whether age appropriate symbolic math training would be as effective at improving math performance as approximate arithmetic training. We also included the ABC Ninja game to provide an active control condition that measures the baseline effects of playing any educational tablet application with an experimenter.

Overall, our design allows for the comparison of approximate arithmetic training to educationally relevant control conditions, and can determine with greater specificity the type of math skills improved due to approximate arithmetic training. Our approximate arithmetic training application, Max's Math Game, has been shown to improve early math skills as measured by the TEMA-3, but ANS acuity correlates with the informal but not formal math questions on the TEMA-3 (Libertus et al., 2013; Park et al., 2016). Moreover, approximate arithmetic training does not involve practice with formal math skills. These facts led us to predict that approximate arithmetic training would improve informal, but not formal, math skills. Conversely, we predicted that 123 Ninja, a numeral identification training application, would improve the formal skill of numeral recognition. Finally, we predicted that letter identification training (ABC Ninja) would not improve either formal or informal math skills, but would improve alphabet knowledge. Finally, consistent with the findings of Park et al. (2016), we predicted no effect of training condition on vocabulary, executive function, or short term memory.

## Methods

### Participants

One hundred and fifty-eight children with a median age of 4.68 (3.27–5.72) were pseudo-randomly assigned to one of three conditions to minimize differences at pretest in age, sex, PPVT, and math score across the groups. Written parental consent was collected in accordance with a protocol accepted by Duke University's Institutional Review Board. Children were drawn from 7 different preschools and we attempted to consent all parents with children aged 3–5 at each preschool location. Five of the 7 preschools were in the North Carolina Pre-K program. This program provides preschool education for children of low socio-economic status. In order to be eligible for this program, parental income must be no more than 75% of the state median income. Eighty-four percent of the participants in our study were enrolled in the NC-PreK program. We obtained detailed demographic data for 86 children. Among this subset of our sample, 26% identified as Hispanic, 63% as not Hispanic, and 11% did not report. Sixty-one percent of the sample identified as African American, 6% as Caucasian, 9% as Asian, American Indian or mixed race, and 24% did not report. Thirty-four percent of the mothers reported a high school degree or some high school, 38% reported a college degree or some college, 16% graduate degree or some graduate school, 3% technical school degree, and 9% chose not to report. Seventeen additional participants were consented but did not complete the study due to a variety of reasons including leaving the school, attending the school on a limited basis, family vacation, or turning 6 years old before testing began. One participant who completed the study was excluded from analysis due to frequent absences and completing the post-test session after an extended winter break (111 days between pre and post tests).

### Procedure

Participants completed a total of 14 experimental sessions: 2 pre-test sessions, 10 training sessions, and 2 post-test sessions. All sessions were administered in a quiet location at the preschool. Each pre and post-test session lasted between 20 and 40 min, and was administered individually. The experimenter who administered pre and post testing was blind to the condition of the child, except for the first 9 participants tested. Pre and post tests consisted of a symbolic math test based on the Number Sense Screener (NSS; Research Edition: Glutting and Jordan, 2012), a short-term memory task, a Stroop interference task, a standard dimensional card sorting task, the Peabody Picture Vocabulary Test 4th Edition (PPVT-4; Dunn and Dunn, 1997), an alphabet knowledge task, and the Give-a-Number task (Wynn, 1990, 1992). Each assessment had two versions and each child was given a different version of the test for pre and post testing with the order of versions counterbalanced across participants. The median time between pre and post test was 27 days. Training sessions occurred in small groups of 3–8 children. During the first training session, children were instructed in how to play the game in detail. After the first training session, children were instructed as needed. Children were monitored for the full 12 min of training to ensure the game was played properly and with full attention. Children wore headphones during training to increase attention to the verbal instructions in each game. Children were rewarded after each experimental session with a sticker of their choice. After all the children in a classroom had completed all 14 sessions, each child received an educational book and building toy, and the classroom was given an additional educational gift chosen by the teacher.

### Training tasks

#### Approximate arithmetic training (max's math)

**Figure 1:**
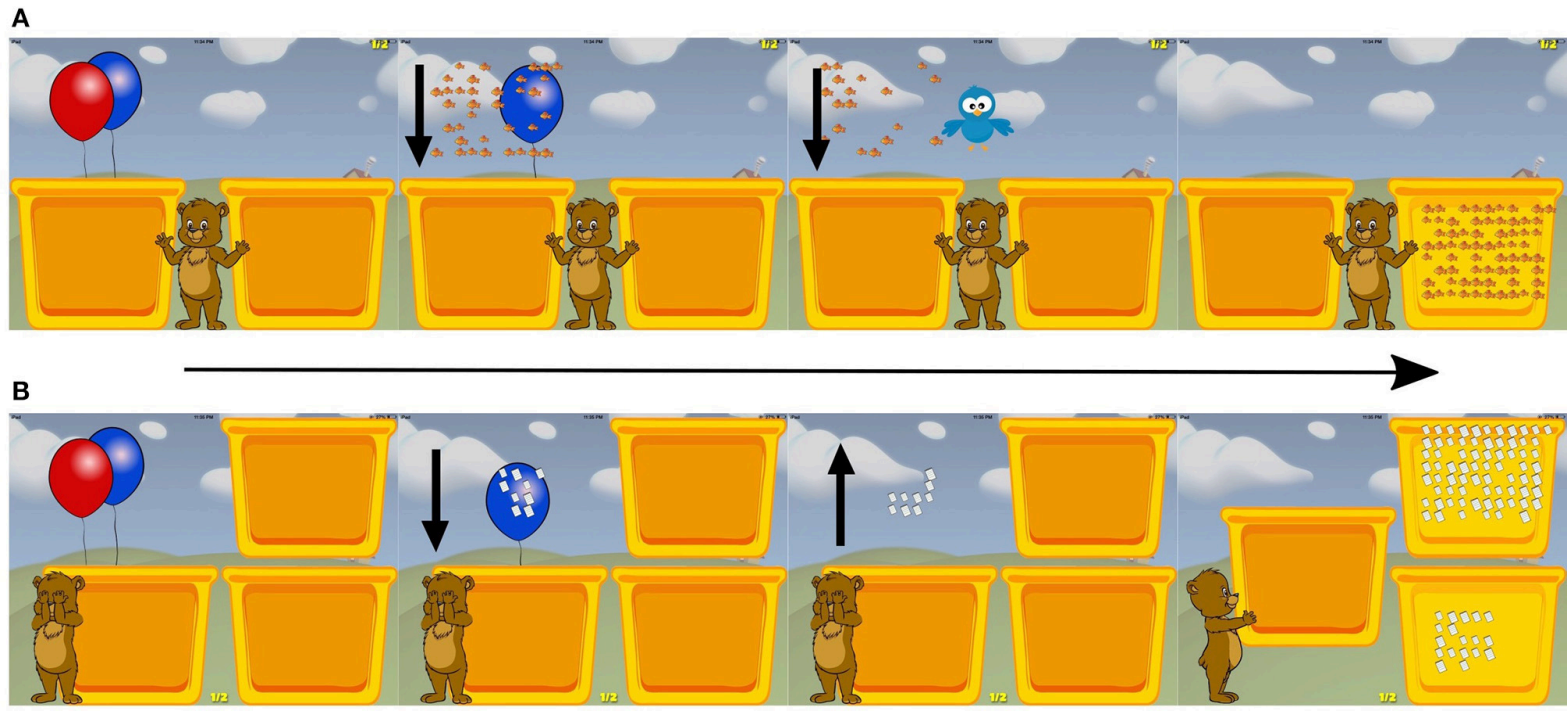
Screenshots of the approximate arithmetic training application (Max's Math Game). **(A)** One Addition comparison trial in Max's Math Game. This is the same approximate arithmetic training game used in Park et al. (2016). The panel farthest to the left is the start of the trial, and the trial ends on the panel farthest to the right where the participant makes their selection. The arrows shown in the middle panels were not displayed during the game. **(B)** One Subtraction comparison trial in Max's Math Game.

A trial began with Max (a cartoon bear) holding a red balloon (Figures 1A,B). Children were instructed to “pop the red balloon,” by touching it, at which point the balloon popped and dropped an array of 4–64 discrete objects (e.g., ears of corn, elephants) into an opaque container. There were four trial types: Addition Comparison, Subtraction Comparison, Matching Addition, and Matching Subtraction. During the addition trials, a second blue balloon popped and dropped more of the objects into the same container. On subtraction trials, the blue balloon popped to reveal a bird that flew in and removed a portion of the original set of objects from the container and off the screen. On comparison trials, children compared the remembered sum or difference to a new target array that appeared in a second container to the right, and were instructed to choose the container that held more items. On matching trials, children were shown two new target arrays with visible objects, and children picked the container that held the same number of items as the remembered sum or difference. Children were given each of the 4 trial types in separate 10-trial blocks. After two blocks of 10 trials each, a short 45–60 s movie played to maintain attention. On half of the matching trials the container with the smaller number of items was the correct choice. Children completed as many trials as possible in 12 min. The median number of trials completed per session was 39 (standard deviation of 5.3) or about 1 block of each trial type per 12-min training session.

Difficulty was titrated based on performance by manipulating the ratio of the target array to the remembered sum or difference. To do this we varied the numerical distance between the target and the alternative in a log-base 2 scale (the log difference level). The game began with a log difference level of 2 (the ratio between the arrays was 1:2^2^ or 1:4). For example, if the target was 20, the alternative was either (20^*^4) or (20/4). The log difference level changed based on the child's average accuracy in a block of 10 trials. If the average accuracy was <60% the log difference level increased by one of the values randomly chosen from [0.08, 0.09, 0.10, 0.11, 0.12] for the next block. If the average accuracy was between 65 and 80% the log difference level stayed the same. If the average accuracy for the block was greater or equal to 80%, the log difference level decreased by one of the values randomly chosen from [0.13, 0.14, 0.15, 0.16, 0.17] for the next block. Each trial type was titrated separately. The log difference level was never allowed to exceed 2.

#### 123 Ninja—numeral identification training

123 Ninja is a commercial educational application found the on the Apple App Store, and is made by Alligator Apps (alligatorapps.com). In this game children hear a number word, as two or three numerals appear on the screen. Children must swipe the numeral corresponding to the number word they hear with their finger. If they correctly identify the numeral, the game makes a sound indicating a correct response, and the bar at the top of the screen begins to fill up. Once the bar is filled completely, the child is awarded a star, which then appears at the top of the screen throughout the rest of the session. If a child swipes the incorrect numeral, a popping noise is made and the incorrectly swiped numeral turns gray. The same number is repeated until a child swipes the correct numeral. The task was not titrated for difficulty. Children completed as many trials as they could in the 12-min training session. The numerals ranged from 0 to 19. Each number was identified ~3 times over the course of 1 training session.

#### ABC ninja—letter identification training

ABC Ninja is made by the same app developer, Alligator Apps. It is exactly the same as 123 Ninja, except that letters appear on the screen instead of numerals. All capital letters A-Z were used.

### Pre and post tests

#### Informal math test

As a measure of symbolic math we modified the NSS to make it appropriate for preschoolers (NSS; Research Edition: Glutting and Jordan, 2012). We used this measure instead of the TEMA-3, because the NSS is divided into question types. This allowed us to assess performance on informal and formal math questions separately. Our test included five informal problem types: Counting, Symbolic Number Comparisons, Nonverbal Calculation, Arithmetic Story Problems, and Simple Arithmetic Problems. The problems used in the NSS were expanded, the wording of some problems changed, and a B version of the test was created to make the test appropriate for preschool aged children and our research questions. The counting section included counting items on a page, and verbally counting as high as possible. The symbolic number comparisons section included questions such as “Which is bigger or more, 6 or 8?” and “What number comes right after 7?” with visual displays of the numerals. In nonverbal calculation, children were shown 1–4 tokens that were subsequently moved under an opaque paper flashcard in an arithmetic operation. For example, on one trial a child was first shown 3 tokens which were then hidden under the flashcard. Then, the child was shown 2 new tokens which were then hidden under the same flashcard. The child had to put the exact same number of tokens under their own flashcard to match the answer to the addition or subtraction problem modeled by the experimenter. In the arithmetic story problems section there were questions such as “You have 4 pennies. I give you 2 more pennies. How many pennies do you have altogether?” The simple arithmetic problem section included questions such as “How much is 7 take away 4?” and “How much is 2 and 1 altogether?” while the numerals in the question appeared on a book in front of the child. There were 28 total questions, and performance was measured as the total number of correctly answered questions. The published test re-retest reliability score for the NSS is 0.81 for kindergarteners measured a month apart. Additionally, we correlated pre and post test scores of all subjects to get a proxy measure of reliability in our sample. The Pearson correlation coefficient between the pre and post test scores of the informal math test was 0.65, indicating reasonable reliability.

#### Formal math test

The formal section of the math test consisted of 8 numeral identification questions. Children were shown a numeral and asked “What number is this?” Performance was measured as the total number of correct answers. The correlation coefficient between pre and post test formal math score was 0.81, indicating high reliability.

#### Number word knowledge

The cardinality section was the give-a-number task (Wynn, 1990, 1992). In this task, each child was presented with a plate of fish, introduced to a stuffed dinosaur, and told the animal was hungry. The experimenter then asked “Can you give the dinosaur one fish?” Once the child placed fish on the plate she/he was asked “Is that one fish?” Children were allowed to fix their responses, and there was no time limit. If successful, the child was then asked to give the dinosaur two fish and given time to correct their answer. On each subsequent trial children were asked to give the dinosaur N+1 (if successful) or N−1 (if unsuccessful) fish. No feedback was provided. Trials continued until there were 2 successes at a given N and two failures at N−1, with *N* = 6 as the maximum value requested. Children were categorized by knower level defined as the highest number they could successfully produce. The correlation coefficient between pretest knower level and post-test knower level was 0.77.

#### Short term memory task: letter span

Children listened as the experimenter read a string of letters. The child was then asked to repeat the letters back in the same order. There were 6 blocks of 5 trials each. In each successive block the string of letters increased by one letter, so that the first block contained two letter strings and the last block contained seven letters. Children continued until they missed 3 or more trials in one block. For the A and B versions of the task the same letters were used, but in a different order. Only monosyllabic letters were used, and letters with similar sounds (e.g., v and b) were excluded. We used this short term memory task for consistency with the Park et al. (2016) experiment. However, it is important to note this is a measure of verbal short term memory, not visual short term memory. One participant in the ABC Ninja condition did not complete this task. Performance was measured as the total number of successful trials. The correlation coefficient between pretest and post-test short term memory score was 0.75.

#### Executive function: standard dimensional change card sort and stroop interference task

To measure executive function, we used two tasks and created a composite score to increase reliability and validity in the measurement (Moreau et al., 2016). The scores from each task were averaged to create a unit-weighted composite score where both tasks were weighted equally. The first task was a Standard Dimensional Change Card Sort. In this task, children must sort a set of objects two different ways: by object category and by color. First, children were given a stack of 10 cards with black and white images of fish and birds. Two boxes were placed in front of them, one marked with a picture of a black fish and the other with a picture of a white bird. The child was then asked to sort the cards by shape (fish or bird). The number of cards sorted correctly and time it took to complete the task was recorded. Next, the child was shown how to sort the cards by color with three example cards, and then was asked to sort the 10 cards by color (white or black). Again, the number of cards sorted correctly and the time it took to complete the task was recorded. For version B, the cards had white or black ships or planes. For version A, the cards were fish or bird in a 5:5 ratio, and were black or white in a 6:4 ratio. For version B, the cards were plane or ship in a 6:4 ratio and black or white in a 5:5 ratio. One participant in the 123 Ninja condition did not complete this task. A composite score of the total number of correctly sorted cards divided by the total time to sort all the cards during the second sorting was used to measure performance. The second task was a Stroop Interference task. Children were shown images of either a cat or a dog one at a time on a flashcard. In the first part of the task, children are asked to name off each image as soon as they see it, and the experimenter marks if they are correct or incorrect. Total time naming the images was also recorded. For the second part of the task, the child is asked to say the opposite animal. For example, if they see a cat, they should say dog and vice versa. Again, responses were scored as correct or incorrect based on the child's first response, and the total time naming the images was recorded. Each part of the task contained 16 images with a ratio of 1:1 for each image type. For version B children were shown images of ducks and cows. This task was adapted from the Gerstadt et al. (1994) day/night task. One participant in the ABC Ninja condition did not complete this task. A composite score of the total number of correctly named animals divided by the total time to name all the animals when the animal names were reversed was used to measure performance. The correlation coefficient between pre and post test executive function composite score was 0.69, indicating reasonable reliability.

#### Pearson's picture vocabulary test

Vocabulary was assessed using the PPVT-4 (PPVT-4; Dunn and Dunn, 1997). A child is shown a booklet with four images on each page. The experimenter reads a word out loud and the child is asked to point to the corresponding image. The task continued until the child answered incorrectly on 10 or more words in a block. Scores were normalized with a standard score of 100. The reported standardized test-retest reliability for the PPVT is high, with a correlation coefficient of 0.91–0.94 within the age range of our participants. In our sample, the correlation coefficient between pre and post test PPVT score was 0.75, indicating reasonable reliability.

#### Alphabet knowledge

Children were shown each of the 26 letters of the alphabet on a flashcard. All letters presented were uppercase letters printed in Chalkboard SE font. Two different orders were used and the order was counterbalanced across children in each condition. Children were asked to name each letter as it was presented, and their responses were recorded. Performance was measured as the total number of correctly identified letters. The correlation coefficient between pre and post alphabet knowledge score was 0.94, indicating high reliability.

## Results

### Training performance

Participants in the approximate arithmetic training condition showed a consistent decrease in log difference level, indicated by the negative correlation between log difference level and trial across all training sessions (*r* = −0.99, *p* < 0.0001). The Ninja games were commercial applications and were not intended for data collection, and so measures of performance over training were less precise. At the end of each training session, the applications returned how many times the child swiped the correct symbol when it appeared. This measure indicated that across all sessions children swiped the correct numeral 63% of the time in the 123 Ninja condition, and the correct letter 67% of the time in the ABC Ninja condition. There was no evidence of a change in the number of correctly swiped letters from the first to last day of training in either Ninja condition (123 Ninja, *t* = 1.26, *p* = 0.21; ABC Ninja, *t* = 0.95, *p* = 0.34).

### Analysis of transfer effects

Pre and post test scores for each measure are presented in Table 1. At pretest, there was no significant difference in pretest score by training condition [math composite, *F*_(2, 154)_ = 0.162, *p* = 0.85; informal math, *F*_(2, 153)_ = 0.098, *p* = 0.91; formal math *F*_(2, 153)_ = 0.235, *p* = 0.79; PPVT, *F*_(2, 153)_ = 0.206, *p* = 0.81; executive function, *F*_(2, 150)_ = 0.063, *p* = 0.94; short term memory *F*_(2, 151)_ = 1.05, *p* = 0.35; alphabet knowledge, *F*_(2, 154)_ = 2.20, *p* = 0.12; Give-a-number, Kruskal–Wallis χ^2^ = 0.710, *df* = 2, *p* = 0.70]. To examine change in performance from pretest to posttest gain scores were calculated for each participant for each measure. The standardized gain score for each measure was calculated by subtracting pretest score from posttest score and then dividing the gain scores by the standard deviation of the pretest scores for that measure. This allowed a comparison of gain scores across different measures. We excluded any standardized gain score when the value was smaller than Q1–3 × IQR or larger than Q3 + 3 × IQR (where Q1 is the first quartile, Q3 is the third quartile and IQR is in the interquartile range). This procedure removed 10 gain scores out of 1,099 data points (<1% of the data). Outlier gain scores included 1 PPVT gain score, 4 composite executive function scores, 1 informal math score, 1 formal math score, and 3 short term memory scores. Outliers were distributed across all 3 training conditions.

**Table 1:** Mean pre and post test scores (and standard deviations) for each training condition.

	***N***	**Informal math**		**Formal math**		**PPVT**		**Executive function composite**		**Short term memory**		**Alphabet knowledge**		**Number word knowledge**	
		**Pre**	**Post**	**Pre**	**Post**	**Pre**	**Post**	**Pre**	**Post**	**Pre**	**Post**	**Pre**	**Post**	**Pre**	**Post**
Approximate arithmetic	53	10.673(3.36)	11.531(3.46)	2.735(1.81)	2.980(1.59)	96.837(15.56)	100.53(14.56)	0.104(0.74)	0.032(0.85)	10.837(3.61)	11.347(3.21)	14.143(9.50)	14.592(9.46)	4.347(1.82)	4.490(1.71)
ABC Ninja	52	10.755(4.04)	11.429(4.10)	2.653(1.91)	2.959(2.03)	97.429(16.43)	100.00(14.88)	0.081(0.84)	0.076(0.80)	11.592(3.98)	12.367(3.95)	13.204(8.98)	15.224(8.80)	3.939(2.04)	4.469(1.93)
123 Ninja	52	10.592(3.52)	11.163(3.83)	2.510(1.95)	2.592(1.77)	95.000(17.38)	95.429(17.59)	0.085(0.76)	0.065(0.74)	10.510(3.31)	11.673(3.47)	10.796(9.87)	11.878(9.77)	4.041(1.98)	4.041(2.06)

Transfer effects were first analyzed with an ANOVA to compare average gain score by condition for each pre/post test. This analysis collapsed across age, gender, and socioeconomic status. Contrary to our main prediction there was no significant difference in math gain score as a function of condition [Formal Math *F*_(2, 153)_ = 0.956, *p* = 0.39; Informal Math *F*_(2, 153)_ = 0.133, *p* = 0.88]. There was also no significant effect of condition on gain score for the PPVT-4 [*F*_(2, 153)_ = 0.652, *p* = 0.52], short term memory [*F*_(2, 151)_ = 0.600, *p* = 0.55], or executive function [*F*_(2, 150)_ = 0.272, *p* = 0.76]. A Kruskal–Wallis Test for nonparametric group differences revealed no effect of condition on improved knower level in the Give-a-number task (Kruskal–Wallis χ^2^ = 4.02, *df* = 2, *p* = 0.13). There was, however, a significant effect of condition for the alphabet knowledge test [*F*_(2, 154)_ = 2.97, *p* = 0.05]. Pairwise comparisons with the Holm correction indicate a significant difference between participants in the approximate arithmetic condition and ABC Ninja (*p* = 0.05), but not between 123 Ninja and ABC Ninja (*p* = 0.24) or between approximate arithmetic and 123 Ninja (*p* = 0.41). Thus, children in the ABC Ninja condition gained more knowledge of the alphabet compared to children in the approximate arithmetic condition, but not significantly more than children in the 123 Ninja condition.

We next examined whether socioeconomic status, age, math ability level, gender, experimental design factors, or training condition influenced performance on the informal and formal math test. We conducted two separate variable selection procedures to select a model that best predicted posttest informal and formal math score. We included the following variables in both variable selection procedures: pretest math composite score, training condition, training condition by pretest math composite score interaction, gender, age, whether or not the child was enrolled in NC-PreK (a proxy for SES), the version of math test the subject took at pretest (A or B), and the number of days between the pre and post test. First, stepwise model selection was performed to minimize AIC using the MASS package “stepAIC” command in R (Venables and Ripley, 2002). Both the addition and deletion of variables were allowed with this stepwise procedure. The final model selected using a minimal AIC criteria with the informal math test as the outcome included the predictors of pretest math score, approximate arithmetic condition, pretest math score by approximate arithmetic condition interaction, gender, age, and enrollment in NC-PreK (AIC = 94.64). To confirm this model, all subsets regression using the C_p_ statistic was conducted with the leaps package “leaps” command in R (Lumley and Miller, 2009). Using this analysis, the model derived from the minimal AIC procedure had a C_p_ statistic of 4.85 with 6 predictors, indicating a slight overfitting. The model that included both the main effect of 123 Ninja and pretest math score by 123 Ninja interaction as well as all the predictors from the previous model was a better fit (Table 2; C_p_ = 8.82, with 8 predictors plus the intercept). The model derived from the all subsets regression procedure with pretest math score, condition main effects, pretest score by condition interactions, age, gender, and NC-PreK enrollment as regressors is presented in Table 2.

**Table 2:** Summary of regression analyses for variables derived from model selection procedures predicting informal and formal math scores (*N* = 157).

**Variable**	**Informal math**			**Formal math**		
	***B***	***SE β***	**β**	***B***	***SE β***	**β**
Intercept	−1.54	0.54	−1.54^**^	0.955	0.08	0.067
Pretest score	0.670	0.10	0.643^***^	0.762	0.05	0.811^***^
123 Ninja condition	0.177	0.14	−0.014	−0.300	0.12	−0.168
Approximate arithmetic condition	4.39	0.14	0.111	−0.064	0.11	−0.036
Pretest score by 123 Ninja condition interaction	−0.021	0.14	−0.020	–	–	–
Pretest score by approximate arithmetic condition interaction	−0.371	0.14	−0.356^*^	–	–	–
Gender	−1.15	0.12	−0.307^*^	–	–	–
Age (in days)	0.004	0.0003	0.001^***^	–	–	–
NC-PreK enrollment	−1.43	0.18	−0.380^*^	–	–	–
*R^2^*	0.50			0.66		
*F*	18.26^***^			100.5^***^		

*All estimates are relative to the performance of the children in ABC Ninja condition*.

*** *p < 0.001*,

** *p < 0.01*,

* *p < 0.05 Gender was coded with girls indicated with a 1, and boys indicated by a 0. Enrollment in NC-PreK was coded as a 1, and private school enrollment with a 0. The age variable was coded in days*.

In Table 2, all estimates are relative to the non-math control condition of ABC Ninja. Math scores were Z-scored so that estimates can be interpreted as effect sizes in terms of standard deviations. First, and most crucial to our main hypothesis, the interaction term between pretest math score and the approximate arithmetic training condition was significant [*F*_(2, 149)_ = 6.48, *p* = 0.01], while the main effect of the 123 Ninja condition [*F*_(2, 149)_ = 0.081, *p* = 0.78], and the interaction of math pretest score and 123 Ninja condition [*F*_(2, 148)_ = 0.021, *p* = 0.88] was not significant. The disordinal interaction between pretest math score and the approximate arithmetic condition indicates that for children with low pretest math scores, approximate arithmetic training resulted in greater math performance at posttest than the ABC Ninja training condition. In contrast, for participants with high math scores, training with ABC Ninja resulted in better math performance.

In addition to a significant interaction of pretest math score and condition, there are also main effects of gender, age, and SES on posttest symbolic math score. On average with all else held constant, girls scored 0.307 standard deviations worse on the symbolic math post-test [*F*_(2, 149)_ = 7.59, *p* = 0.007] compared to boys. Age of the child was also a significant predictor of posttest symbolic math score, which is expected in a non-standardized math test. When all else was held constant a child answered 0.001 standard deviations better for every day they aged [*F*_(2, 149)_ = 13.18, *p* = 0.0004]. Thus on average a child answered 0.365 standard deviations better for every year of age. Finally, whether or not the child was enrolled in state funded preschool was also a significant predictor of post-test math score. On average with all else held constant, children in state funded preschools answered 0.380 standard deviations worse compared to students funded by private tuition [*F*_(2, 149)_ = 6.17, *p* = 0.01]. Overall, this analysis reveals that in addition to pretest math score and condition, gender, enrollment in state funded preschool, and age also impacted children's math test performance after training. Important to the goal of this experiment, accounting for the variance in informal math score due to SES, gender, and age revealed an effect of training condition for the low math scoring participants consistent with our hypothesis that approximate arithmetic training improves informal math ability.

We then ran both model selection procedures to test whether age, math ability level, SES, gender, or experimental design factors impacted performance on formal math problems. The final model selected using a minimal AIC criteria included the predictors of pretest math score and the 123 Ninja condition (AIC = 165.47). Using the best subsets regression model selection technique, the most parsimonious model with the best C_p_ statistic was the same model derived from the minimal AIC criterion (C_p_ = 2.72, with 2 predictors plus the intercept). In this model, only pretest math score explained significant variance in posttest math score [*F*_(2, 154)_ = 299.76, *p* < 0.001] indicating that there were no effects of condition on formal math gains. The results are unchanged when the approximate arithmetic condition is added to the model, and so the model with this predictor is included in Table 2 for better comparison of performance across all three training conditions. Contrary to our hypothesis that 123 Ninja training would improve formal math skill, this analysis indicated no effect of condition on formal math test performance.

### Analysis of transfer effects among low math achieving participants

Our central hypothesis was that children in the approximate arithmetic training condition would improve their informal math skill significantly more than children in the symbol identification training conditions. Contrary to this prediction, we did not find a main effect of condition among the full sample of participants. Instead, we found a significant interaction between informal math pretest score and the approximate arithmetic training condition. This interaction indicated that among participants with a low score on the informal math pretest, the approximate arithmetic training group gained more at post-test than participants in the ABC Ninja condition. Based on this finding, we reran the model in Table 2 with data only from the participants who scored in the lower half of math pretest scores on all measures of the math test (*N* = 87)^1^. Demographics of this half of the data in comparison to the full data set are shown in Table 3.

**Table 3:** Demographics of full sample and participants who scored in the low half of pretest math scores.

**Participants**	**Approximate arithmetic**		**123 Ninja**		**ABC Ninja**	
	**All**	**Low math**	**All**	**Low math**	**All**	**Low math**
*N*	53	27	52	29	52	31
Gender (males)	27	10	26	14	26	18
Age in years [mean (*sd*)]	4.57 (0.61)	4.37 (0.62)	4.61 (0.52)	4.48 (0.51)	4.58 (0.56)	4.41 (0.59)
Enrollment in NC-PreK	45	24	44	25	43	25

**Figure 2:**
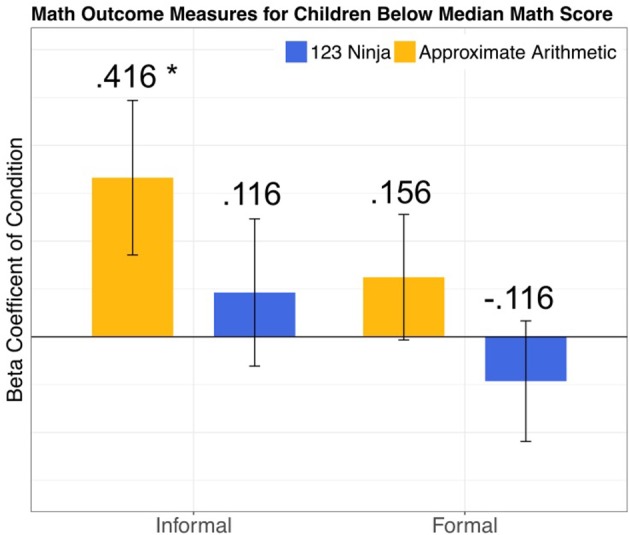
Colored bars depict the impact of the approximate arithmetic and 123 Ninja training conditions on each math outcome measure. Error bars represent the standard error of the coefficients. Coefficient estimates are relative to the performance of children in the ABC Ninja training condition. Coefficient estimates are Z-scored so they can be interpreted as effects sizes in terms of standard deviations. Asterisks reflect rejection of the null hypothesis of no difference compared to the ABC Ninja training control group. These coefficients are taken from the models reported in Table 4.

Critical to our central hypothesis that approximate arithmetic training improves informal math ability, there was a significant main effect of the approximate arithmetic condition among participants with a low pretest math score [Table 4 and Figure 2; *F*_(2, 81)_ = 4.24, *p* = 0.04]. This indicates that for children with low math skills, approximate arithmetic training resulted in higher post-test informal math scores than participants in the ABC Ninja training condition. As expected, the interaction between pretest math score and condition was no longer significant among this subset of participants [*F*_(2, 80)_ = 0.230, *p* = 0.81]. The main effect of the 123 Ninja condition was also not significant [*F*_(2, 81)_ = 0.363, *p* = 0.55]. Thus, there was no effect of the 123 Ninja training condition compared to the ABC Ninja condition on post-test informal math score. Overall, these results are in line with our original hypothesis that approximate arithmetic training improves informal math skills compared to the ABC Ninja condition, however, this effect is limited to children with low initial math performance.

**Table 4:** Summary of regression analyses for low pretest scoring math participants predicting informal and formal math scores (*N* = 87).

**Variable**	**Informal math**			**Formal math**		
	***B***	***SE β***	**β**	***B***	***SE β***	**β**
Intercept	4.56	0.69	−1.45^*^	0.894	0.12	−0.131
Pretest score	0.131	0.16	0.126	0.651	0.08	0.694^***^
123 Ninja condition	0.434	0.19	0.116	−0.217	0.16	−0.122
Approximate arithmetic condition	1.56	0.20	0.416^*^	0.242	0.16	0.136
Gender	−1.41	0.17	−0.375^*^	–	–	–
Age (in days)	0.003	0.0004	0.0008^†^	–	–	–
NC-PreK enrollment	−0.916	0.25	−0.244	–	–	–
*R^2^*		0.12			0.48
*F*		1.863^†^			25.85^***^	

*All estimates are relative to the performance of the children in ABC Ninja condition*.

*** *p < 0.001*,

* *p < 0.05*,

† *p < 0.1 Gender was coded with girls indicated with a 1, and boys indicated by a 0. Enrollment in NC-PreK was coded as a 1, and private school enrollment with a 0. The age variable was coded in days*.

Among children with a low pretest math score, there was also a significant effect of gender [*F*_(2, 81)_ = 4.86, *p* = 0.03] and a marginal effect of age [*F*_(2, 81)_ = 3.49, *p* = 0.07] on informal math post-test score, but there was no longer an effect of SES [*F*_(2, 81)_ = 0.966, *p* = 0.33]. The gender effect indicates the girls scored 0.375 standard deviations worse on the symbolic math posttest compared to boys. The age effect indicates that children scored 0.0008 standard deviations better for every day they aged, or 0.292 standard deviations for every year they aged. These effects are similar to the gender and age effects found for the full sample of participants.

Finally, similar to the results including the full sample of participants, there was no effect of condition on post-test formal math score, but there was a significant effect of pretest formal math score [Table 4; *F*_(2, 84)_ = 73.178, *p* < 0.001]. This result indicates that training condition had no impact on formal math ability. When the effects of age, gender, and enrollment in state funded preschool on formal math score are controlled, there is still no effect of condition on formal math score [Figure 2; Approximate Arithmetic *F*_(2, 81)_ = 0.901, *p* = 0.35; 123 Ninja *F*_(2, 81)_ = 0.541, *p* = 0.46]. Also consistent with the findings for the full sample of participants, children in approximate arithmetic condition with a low initial math score did not improve on measures of vocabulary [*F*_(2, 81)_ = 0.000, *p* = 0.98], short term memory [*F*_(2, 78)_ = 0.001, *p* = 0.92], executive function [*F*_(2, 81)_ = 1.55, *p* = 0.22], or number word knowledge [*F*_(2, 81)_ = 0.175, *p* = 0.68] when controlling for effects of age, gender, and state funded preschool enrollment. Children in the 123 Ninja condition also did not improve on these measures, however, they did perform significantly worse at post-test on the PPVT-4 than children in the ABC Ninja condition [*F*_(2, 81)_ = 6.55, *p* = 0.01]. Consistent with our original hypothesis, low math scoring children in the ABC Ninja condition performed significantly better on letter identification than children in both the approximate arithmetic [*F*_(2, 81)_ = 7.28, *p* = 0.008] and 123 Ninja [*F*_(2, 81)_ = 5.81, *p* = 0.02] conditions at post-test when controlling for pretest score, indicating that ABC Ninja training improved children's letter identification skill. Overall, these results demonstrate the specificity of the approximate arithmetic training effect. Improvements in informal math skill among low math scoring approximate arithmetic participants were not due to increases in short term memory, executive function, vocabulary, or number word knowledge.

## Discussion

Our study was designed to ask whether approximate arithmetic training positively impacts informal, and not formal, math ability in preschool aged children over and above any benefits of two commercially available educational applications. Contrary to our hypothesis, we did not find a benefit of approximate arithmetic training on informal math performance for all participants. Instead, we found that for children with low math scores, approximate arithmetic training significantly improved informal symbolic math performance compared to training that focused on letter knowledge. While unexpected, this finding is consistent with previous research that has found the correlation between ANS acuity and math performance only among children who scored poorly on a math assessment (Bonny and Lourenco, 2013; Purpura and Logan, 2015). Consistent with our hypothesis, the positive effect of approximate arithmetic training was restricted to informal, and not formal, math abilities. We found no effect of training condition on formal math abilities, however, ABC Ninja training was effective at improving alphabet knowledge.

Previous research with our approximate arithmetic training application, Max's Math, found that approximate arithmetic training improved the math skills of preschool children across the range of math performance (Park et al., 2016). It is important to note that the magnitude of the math standardized gain score for our approximate arithmetic training condition with all participants (0.251 with standard error 0.137) is within the standard error found for the math standardized gain score of the approximate arithmetic training condition in Park et al. (2016; 0.307 with standard error 0.070). In Park et al. (2016), the math gain score for the approximate arithmetic training group was significantly different than the math gain score for picture memory control training condition, whereas in our study among the full sample of participants the math gain scores for the symbol identification control conditions were not significantly different from the approximate arithmetic training group. It is possible that the commercially available symbol identification training games used in our study were more engaging than the picture-memory control condition used in Park et al. (2016).

A major difference between Park et al. (2016) and the current study, was that Park et al. (2016) used the TEMA-3 as an outcome measure whereas we used a modified version of the NSS. The standardized gain score for the approximate arithmetic condition in the previous study was slightly, if not significantly, higher than the gain score for the approximate arithmetic condition in the current study. The TEMA-3 may be more sensitive to the math abilities improved by approximate arithmetic training than the math measure in the current study. Additionally, the TEMA-3 is standardized to be age appropriate for children 3–9 years old, whereas the NSS was developed for children in kindergarten to 1st grade. It is possible that despite our attempt to modify the measure it was not age appropriate for preschool children.

Surprisingly, despite the fact that the 123 Ninja task was designed to teach the association between numerals and number words, children trained in this condition did not improve on our formal math test of numeral identification. In contrast, children in the ABC Ninja condition did improve in their alphabet knowledge at post-test significantly more than children in the approximate arithmetic condition. A strong possibility is that our measure of numeral identification was not as sensitive as our measure of alphabet knowledge. The numeral identification test included double-digit numbers that were not explicitly trained, while our measure of alphabet knowledge included all of the letters of the alphabet. This design resulted in a greater overlap between training and test for the ABC Ninja condition compared to the 123 Ninja condition. It is likely that with a better matched numeral identification measure 123 Ninja training would also be effective at improving numeral identification.

Another aspect of our hypothesis was that children in the approximate arithmetic condition would improve selectively on informal symbolic math skills, and not on tests of short term memory or executive function. Children in the approximate arithmetic condition with a low pretest math score improved selectively on informal math skills, and not on our pre/post test measures of executive function, short term memory, vocabulary, or number word knowledge skills. This finding suggests that improvement on informal math skills was due to the manipulation of non-symbolic quantity, and not due to improvements on short term memory or executive function skills or to differences in number word knowledge or vocabulary.

We also found that both gender and SES influenced children's performance on the symbolic math test. Overall boys performed better on our math assessment than girls. Gender differences in performance were not reported for the NSS, the standardized math test our math measure was based upon (Research Edition: Glutting and Jordan, 2012), although work with an earlier version of the test did find a small effect of gender in the same direction as our effect (Jordan et al., 2006). We also found an effect of socio-economic status on post-test math scores consistent with previous findings (Starkey et al., 2004; Jordan et al., 2006, 2007). Indeed, Park et al. (2016) found that approximate arithmetic training was particularly effective among low income children. Our study offers more evidence that socio-economic status impacts early math learning.

In summary, consistent with our original hypothesis, approximate arithmetic training improved informal math skills significantly more than training with letter identification, however, this effect was restricted only to children with low math skill. We found a significant interaction between pretest math ability and training condition, such that low math performance participants benefitted more from approximate arithmetic training, while high math performance participants had higher post-test informal math scores after symbol identification training. Among low scoring math participants, there was a main effect of higher post-test math scores among children in the approximate arithmetic condition compared to children who trained on letter identification. As predicted, this effect was restricted to informal, and not formal, math skills. Overall, our results support the conclusion that approximate arithmetic training may be especially effective for children with low math skills, while children with a high level of math skill benefit more from symbolic training. Our study is also consistent with the general conclusion that training on educationally focused tablet applications can be effective in teaching children early academic skills.

Additional research is necessary to identify the precise conditions under which approximate arithmetic training benefits children's math learning. While we were able to demonstrate that approximate arithmetic benefits informal math ability, this category still encompasses a wide array of math skills. Future studies should implement a larger battery of informal math questions to identify the specific math skills that benefit the most from approximate arithmetic training. Another open question is the level of math skill the child brings to the table when beginning training with approximate arithmetic. Our findings suggest that approximate arithmetic may be especially beneficial for children with low math ability. Future work should explore how math ability and factors that can broadly effect math ability, such as socio-economic status and age, interact to influence the effectiveness of intervention. Finally, our research supports the idea that approximate arithmetic training could be a useful addition to an early math curriculum, but further research is needed to understand the best way to integrate non-symbolic and approximate arithmetic into early math education. A recent convergence of work supporting the effectiveness of approximate arithmetic training, including the current study, suggests this would be a useful endeavor.

## Ethics statement

The study and protocol were reviewed and approved by the Duke University's Institutional Review Board. Written informed consent was obtained from the parents of all participants.

## Author contributions

ES and EB conceived and planned the experiment. ES collected the data and performed the analyses. All authors discussed the results and contributed to the final manuscript.

### Conflict of interest statement

The authors declare that the research was conducted in the absence of any commercial or financial relationships that could be construed as a potential conflict of interest.

## Footnotes

^1^Note that participants who scored at the median pretest math score (*N* = 14) were included in the low pretest math score group.
